# Double muscle innervation using end-to-side neurorrhaphy in rats

**DOI:** 10.1590/S1516-31802012000600004

**Published:** 2013-01-18

**Authors:** Elisangela Jeronymo Stipp-Brambilla, Fausto Viterbo, Daniel Labbé, José Antonio Garbino, Maíra Miranda Bernardelli

**Affiliations:** I MSc, PhD. Biologist, Department of Surgery and Orthopedics, Faculdade de Medicina de Botucatu (FMB), Universidade Estadual Paulista (Unesp), Botucatu, São Paulo, Brazil.; II MD, PhD. Plastic Surgeon, Department of Surgery and Orthopedics, Faculdade de Medicina de Botucatu (FMB), Universidade Estadual Paulista (Unesp), Botucatu, São Paulo, Brazil.; III MD, PhD. Plastic Surgeon, Department of Plastic Surgery, University Hospital, Caen, France.; IV MD, PhD. Chief of Clinical Neurophysiology, Lauro de Souza Lima Institute, Bauru, São Paulo, Brazil.; V Medical Student, Faculdade de Medicina de Botucatu (FMB), Universidade Estadual Paulista (Unesp), Botucatu, São Paulo, Brazil.

**Keywords:** Microsurgery, Sciatic nerve, Tibial nerve, Peroneal nerve, Rats, Microcirurgia, Nervo ciático, Nervo tibial, Nervo fibular, Ratos

## Abstract

**CONTEXT AND OBJECTIVE:**

One of the techniques used for treating facial paralysis is double muscle innervation using end-to-end neurorrhaphy with sectioning of healthy nerves. The aim of this study was to evaluate whether double muscle innervation by means of end-to-side neurorrhaphy could occur, with maintenance of muscle innervation.

**DESIGN AND SETTING:**

Experimental study developed at the Experimental Research Center, Faculdade de Medicina de Botucatu, Unesp.

**METHODS:**

One hundred rats were allocated to five groups as follows: G1, control group; G2, the peroneal nerve was sectioned; G3, the tibial nerve was transected and the proximal stump was end-to-side sutured to the intact peroneal nerve; G4, 120 days after the G3 surgery, the peroneal nerve was sectioned proximally to the neurorrhaphy; G5, 120 days after the G3 surgery, the peroneal and tibial nerves were sectioned proximally to the neurorrhaphy.

**RESULTS:**

One hundred and fifty days after the surgery, G3 did not show any change in tibial muscle weight or muscle fiber diameter, but the axonal fiber diameter in the peroneal nerve distal to the neurorrhaphy had decreased. Although G4 showed atrophy of the cranial tibial muscle 30 days after sectioning the peroneal nerve, the electrophysiological test results and axonal diameter measurement confirmed that muscle reinnervation had occurred.

**CONCLUSION:**

These findings suggest that double muscle innervation did not occur through end-to-side neurorrhaphy; the tibial nerve was not able to maintain muscle innervation after the peroneal nerve had been sectioned, although muscle reinnervation was found to have occurred, 30 days after the peroneal nerve had been sectioned.

## RESUMO

**CONTEXTO E OBJETIVO:**

Uma das técnicas utilizadas para tratamento da paralisia facial é a dupla inervação muscular com neurorrafia término-terminal, seccionando-se nervos sadios. O objetivo deste trabalho foi avaliar a ocorrência de dupla inervação muscular através de neurorrafia término-lateral e a manutenção da inervação.

**TIPO DE ESTUDO E LOCAL:**

Estudo experimental desenvolvido no Centro de Pesquisa Experimental da Faculdade de Medicina de Botucatu, Unesp.

**MÉTODOS:**

Cem ratos foram distribuídos em cinco grupos: G1, controle; G2, secção do nervo fibular; G3, o nervo tibial foi seccionado e o coto proximal suturado na lateral do nervo fibular íntegro; G4, 120 dias após a cirurgia do G3, o nervo fibular foi seccionado proximal à neurorrafia; G5, 120 dias após a cirurgia do G3, os nervos fibular e tibial foram seccionados proximal à neurorrafia.

**RESULTADOS:**

Após 150 dias da cirurgia, não foi observada variação na massa do músculo tibial ou no diâmetro das fibras musculares no G3, porém, houve redução do diâmetro da fibra axonal do nervo fibular distal à neurorrafia. Embora, no G4, tenha ocorrido atrofia do músculo tibial cranial 30 dias após a secção do nervo fibular, os resultados do teste eletrofisiológico e da medida do diâmetro axonal confirmaram a ocorrência de reinervação muscular.

**CONCLUSÃO:**

Estes resultados sugerem que a dupla inervação muscular não ocorreu através da neurorrafia término-lateral; o nervo tibial não foi capaz de manter a inervação muscular após a secção do nervo fibular; contudo, ocorreu reinervação muscular 30 dias após a secção do nervo fibular.

## INTRODUCTION

Facial paralysis compromises the facial mimetic muscles, which are of great importance for communication, since they are responsible for expressing feelings and emotion. It causes facial disfigurement, which may impair socialization by affecting such individuals’ social lives, as well as causing considerable psychological damage to both adults and children.^1^

Among the several techniques used for treating facial paralysis are transfacial nerve grafting^2^ and transposition of the temporal muscle.^3^ Labbé et al.^4^^,^^5^ reported using these two techniques together. Lengthening temporalis myoplasty was performed, consisting of lengthening the temporal muscle from its original location and then suturing its insertion tendon directly onto the orbicular muscle of the mouth. After muscle transposition, cross-face nerve grafting using the sural nerve was performed. One of its ends was sutured to the proximal stump of one of the sectioned branches of the healthy facial nerve. The other end was sutured to the distal stump of one sectioned branches of the deep temporal nerve, by means of end-to-end neurorrhaphy. Using this technique, the temporal muscle was doubly innervated, i.e. by the two remaining original branches and by the facial nerve using the sural nerve graft. Nerve grafting seemed to enable the transposed muscle to have a better facial function, thereby improving the quality of spontaneous smiles.

When sectioning one of the branches of the deep temporal nerve, as reported by Labbé et al.,^4^ denervation of part of the temporal muscle may occur. Reinnervation by the sural nerve graft is likely to occur in these muscle fibers but, because the graft is long, reinnervation may not work properly or may even not occur, thus leading to muscle atrophy and therefore to functional impairment for the patient. One alternative, to avoid sectioning one of the branches of the deep temporal nerve as well as avoiding the risk of muscle atrophy, would be to carry out double muscle innervation by means of end-to-side neurorrhaphy.^6^ The extremity of the sural nerve graft would be sutured to the side of one of the branches of the deep temporal nerve. In this manner, the temporal muscle would not be at risk of losing its strength or becoming denervated.

However, doubts have been raised about whether double muscle innervation by means of end-to-side neurorrhaphy really occurs, i.e. whether the sural nerve graft and the deep temporal nerve would really innervate the temporal muscle simultaneously. On the other hand, grafts by means of end-to-side neurorrhaphy would allow time for axons to regenerate through this, which would make it possible to cut the branch used, proximally to the neurorrhaphy, in a second surgical procedure. This way, the muscle would not become denervated, thus avoiding muscle atrophy.

## OBJECTIVE

This study evaluated whether double muscle innervation by means of end-to-side neurorrhaphy could occur, with maintenance of innervation through this end-to-side neurorrhaphy after sectioning the recipient nerve proximally to the neurorrhaphy.

## MATERIALS AND METHODS

This study was approved by the Research Ethics Committee of Botucatu Medical School (Faculdade de Medicina de Botucatu, FMB), Universidade Estadual Paulista (Unesp).

A total of 100 male Wistar rats, with a mean body weight of 195.6 (± 18.8) g were kept under controlled temperature (25 ± 2 °C), and a light-dark cycle of 12-hour periods. They were kept in appropriate boxes with five animals in each and received water and food *ad libitum*.

**Figure 1.**
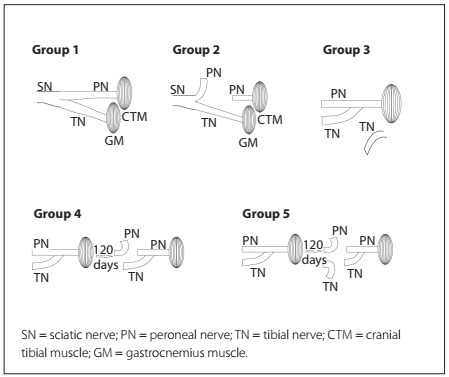
Surgical procedures performed in each group of rats.

After anesthesia using intraperitoneal injections of sodium pentobarbital (30 mg/kg), clipping and aseptic preparation of the right hind limb were performed. The animals were randomly allocated to five experimental groups with 20 animals in each group **(**Figure 1**)**, as follows:

G1: (control group): exposure of the peroneal, tibial and sciatic nerves was performed as the sole procedure; the animals were sacrificed after 120 days.
G2: the peroneal nerve was sectioned and the proximal and distal stumps were inverted through 180 degrees, inserted and fixed into the adjacent musculature to avoid spontaneous reinnervation; these animals were sacrificed after 120 days.
G3: the tibial nerve was sectioned and the proximal stump was sutured to the lateral face of the peroneal nerve, with no opening of the epineural window; these animals were sacrificed after 150 days.
G4: the same procedure as used for G3 was performed. After 120 days, the peroneal nerve was sectioned proximally to the end-to-side neurorrhaphy, and the stumps were inverted through 180 degrees, inserted and fixed into the adjacent musculature. The animals were sacrificed 30 days after the second surgery. The aim of this group was to evaluate whether end-to-side neurorrhaphy would be able to maintain the innervation of the cranial tibial muscle after sectioning of the peroneal nerve.
G5: the same procedure as used for G3 was performed. After 120 days, the peroneal nerve and the tibial nerve were sectioned proximally to the end-to-side neurorrhaphy and the stumps were inverted through 180 degrees, inserted and fixed into the adjacent musculature. The animals were sacrificed 30 days after sectioning. This denervated group was used for comparison with G4.

The neurorrhaphy procedures were performed in the peroneal nerve, about 2 cm distal to the bony sciatic notch, with four simple stitches in the epineurium using 10-0 mononylon thread under a surgical microscope. The incision was sutured with simple stitches using 4-0 mononylon thread.

The animals were sacrificed by means of a lethal dose of sodium pentobarbital intraperitoneally.

### Electrophysiological test

For the electrophysiological test, the electromyography equipment Sapphire II 4ME was used. The room temperature was kept at around 25 °C. After anesthesia, clipping and aseptic preparation, a wide incision was made in the right hind limb, to allow access to the sciatic, peroneal and tibial nerves and the cranial tibial muscle.^7^ The frequency (1 pps), duration (100 µs) and intensity (5.1 millivolts) of the stimulus were fixed. The motor unit action potential was recorded using needle electrodes. The active electrode was placed ventrally to the cranial tibial muscle and the reference electrode was placed near the tendon of insertion of the muscle.

The functional properties of the muscle were evaluated by applying stimuli delivered through bipolar electrodes that had been specially developed for this purpose, in which the cathode and anode were separated by a gap of 2 mm.^7^ The electrode for bipolar stimulation was placed directly on the sciatic nerve in G3, G4 and G5 to enable propagation of electrical impulses through the end-to-side neurorrhaphy. In G1, G2 and G3, the electrophysiological test was performed just before the animals were sacrificed. In G4 and G5, the test was performed on two different occasions: 120 days after the initial procedure, i.e. just before sectioning the nerves; and just before the animals were sacrificed, i.e. 30 days after the nerves had been sectioned.

### Collection of histological material

Segments of the peroneal nerve were collected distally (N1) and proximally (N2) to the end-to-side neurorrhaphy and from the right cranial tibial muscle, and this was done after the electrophysiological test. In G1 and G2, a single sample was collected from a segment of the peroneal nerve in order to make comparisons with the N1 and N2 nerve segments from G3, G4 and G5. In G2, the segment was collected distally to the sectioning. The nerve segments collected were immersed in Karnovsky solution at 4 °C for over 24 hours. The muscles were weighed without their tendons and then frozen in liquid nitrogen for transverse cryostat sectioning.

### Analysis on the cranial tibial muscle

Serial transverse cryostat sections (7 µm) were stained with hematoxylin-eosin and the analyses on the muscle fibers were performed using an optical microscope at a magnification of 200 x. Images were captured using a digital camera attached to the microscope and were saved in a personal computer. The diameters of the muscle fibers were measured using the semi-automatic Sigma Pro Image Analysis software, version 5 (Jandel Scientific Corporation). A total of 50 fibers per muscle were measured.

### Analysis on the nerve segments

The N1 segments (peroneal nerve distal to the end-to-side-neurorrhaphy) and N2 segments (peroneal nerve proximal to the end-to-side-neurorrhaphy) were embedded in resin (Araldite 502) for optical microscopy analysis. Semi-thin transverse sections were stained with osmium tetroxide (1:1) and toluidine blue (1%). Images were captured at 400 x magnification for analyses on nerve fibers, using the same equipment as used for capturing images of muscle fibers. Histological fields (5128 µm²) were selected for quantification of axonal density and the average fiber diameter of all intact axonal fibers. For the field selection, a grid was created covering the entire nerve cross-section, and then every third and fifth fields were analyzed. The same software as described above was used for these procedures. The results were given in mm^2^.

### Statistical analysis

Analysis of variance (Anova) was used to compare all the groups, followed by the Tukey test when a statistical difference was detected. The paired t test was used to compare the results from the electrophysiological tests before nerve sectioning and before the animals were sacrificed (G4 and G5). Student’s t test was used to compare pairs of groups. The significance level was set at P < 0.05 for all analyses.

## RESULTS

### Electrophysiological test

The amplitude and latency results from the electrophysiological tests performed in G4 revealed that there was a decrease in amplitude from 18.7 ± 1.7 mV to 4.9 ± 1.6 mV, over the 30 days after the peroneal nerve had been sectioned. However, no statistical difference was observed when comparing the latency results (1.6 ± 0.1 ms and 1.9 ± 0.3 ms) **(**Table 1**)**.

**Table 1.** Electrophysiological test in groups 4 (G4) and 5 (G5)

Group	Time point	Amplitude (mV)	Latency (ms)
G4	SEC	18.7 ? 1.7^*^	1.6 ? 0.1
	SAC	4.9 ? 1.6	1.9 ? 0.3
G5	SEC	17.4 ? 2^*^	1.7 ? 0.2^*^
	SAC	0.5 ? 0.5	-

SEC = before sectioning of peroneal nerve proximally to the end-to-side neurorrhaphy; SAC = immediately before sacrifice. Paired t test, ^*^statistically significant differences when SEC and SAC were compared in G4 or in G5; P < 0.05.

In G5, the amplitude and latency results from the electrophysiological tests performed before sectioning the peroneal and tibial nerves and 30 days afterwards, just before the animals were sacrificed, showed that a significant decrease in amplitude occurred after sectioning had been performed, from 17.4 ± 2.0 mV to 0.5 ± 0.5 mV. The latency values ranged from 1.7 ± 0.2 ms before sectioning to absence of latency after the peroneal and tibial nerves had been sectioned **(**Table 1**)**.

When all the groups were compared with each another, the amplitude in G3 (21.5 ± 1.9 mV) was lower than that in G1 (29.1 ± 1.8 mV). Thirty days after the peroneal nerve had been sectioned, G4 (4.9 ± 1.6 mV) and G5 (0.5 ± 0.5 mV) were not significantly different from G2 (0.0 mV) **(**Table 2**)**.

**Table 2.** Electrophysiological test in groups 1 (G1) to 5 (G5)

Group	Amplitude (mV)	Latency (ms)
G1	29.1 ? 1.8 a	1.6 ? 0.1 a
G2	0 b	- b
G3	21.5 ? 1.9 c	1.8 ? 0.04 a
G4	4.9 ± 1.6 b	1.9 ± 0.3 a
G5	0.5 ? 0.5 b	- b

Analysis of variance (ANOVA) followed by the Tukey test. The letters a, b and c represent the statistical results. Different letters show statistically significant differences; P < 0.05.

Absence of latency was observed in G2 and G5, which were denervated groups. No significant differences were observed between G1 (1.6 ± 0.1 ms), G3 (1.8 ± 0.04 ms) and G4 (1.9 ± 0.3 ms) **(**Table 2**)**.

### Weight of the right cranial tibial muscle

No statistically significant difference was observed between G3 (0.885 ± 0.04 g) and G1 (0.897 ± 0.02 g) regarding the weight of the cranial tibial muscle. No statistically significant difference was observed between G4 (0.431 ± 0.05 g) and G5 (0.345 ± 0.04 g). The lowest weight value was observed in G2 (0.187 ± 0.02)**(**Table 3**)**.

**Table 3.** Analysis of the right cranial tibial muscle in groups 1 (G1) to 5 (G5)

Group	WCTM (g)	MDMF (µm)
G1	0.897 ? 0.02 a	50.1 ? 2.4 a
G2	0.187 ? 0.03 b	18.5 ? 1.2 b
G3	0.885 ? 0.04 a	51.1 ? 2.3 a
G4	0.431 ? 0.05 c	36.6 ? 2.5 c
G5	0.345 ? 0.04 c	28.6 ? 0.8 c

WCTM = weight of the right cranial tibial muscle; MDMF = minimum diameter of the muscle fiber. Analysis of variance (ANOVA) followed by the Tukey test. The letters a, b and c represent the statistical results. Different letters show statistically significant differences; P < 0.05.

### Diameter of muscle fibers

No statistically significant difference was observed between G3 (51.1 ± 2.3 µm) and G1 (50.1 ± 2.4 µm). Likewise, no difference was observed between G4 (36.6 ± 2.5 µm) and G5 (28.6 ± 0.8 µm). The muscle fiber diameter was also observed in G2 (18.5 ± 1.2 µm) **(**Table 3**)**.

### Axonal density

When N1 nerve segments were compared, no difference was observed between G3 (15 ± 2 axons/mm^2^) and G1 (13 ± 2 axons mm^2^). In addition, no difference was observed between G4 (4 ± 1 axons/mm^2^) and G5 (5 ± 0.5 axons/mm^2^) **(**Table 4**)**.

**Table 4.** Analysis of the nerve segment

Group	AD-N1 (axon/mm^2^)	MAD-N1 (µm)	AD-N2 (axon/mm^2^)	AMD-N2 (µm)
G1	13 ? 2 a	3.1 ? 0.3 a	13 ? 2 a	3.1 ? 0.3 a
G2	1 ? 0.5 b	0.2 ? 0.1 b	1 ? 0.5 b	0.2 ? 0.1 b
G3	15 ? 2 a	2.2 ? 0.2 c	10 ? 1 a	3.1 ? 0.1 a
G4	4 ? 1 c	1.2 ? 0.1 d	5 ? 1 c	1.0 ? 0.1 c
G5	5 ? 0.5 c	1.1 ? 0.2 d	3 ? 1 d	1.6 ? 0.1 c

AD-N1 = axonal density in segment of peroneal nerve distal to neurorrhaphy; AMD-N1 = minimum axonal diameter in segment of peroneal nerve distal to neurorrhaphy; AD-N2 = axonal density in segment of peroneal nerve proximal to neurorrhaphy; AMD-N2 = minimum axonal diameter in segment of peroneal nerve proximal to neurorrhaphy. Analysis of variance (ANOVA) followed by the Tukey test. The letters a, b, c and d represent the statistical results. Different letters show statistically significant differences; P < 0.05.

When N2 nerve segments were compared, no difference was observed between G3 (10 ± 1 axons/mm^2^) and G1 (13 ± 2 axons mm^2^). G5 (3 ± 1 axons/mm^2^) presented lower axonal density than that of G4 (5 ± 1 axons/mm^2^), but was higher than that of G2 (1 ± 0.5 axons/mm^2^) **(**Table 4**)**.

Axonal density was compared between the N1 and N2 nerve segments of G3. The axonal density in the N1 nerve segment (15 ± 2 axons/mm^2^) was higher than that of the N2 nerve segment (10 ± 1 axons/mm^2^) **(**Table 4**)**.

### Axonal diameter

In the N1 nerve segment, the axonal diameter was smaller (2.2 ± 0.2 µm) in G3 than in G1 (3.1 ± 0.3 µm). No statistically significant difference was observed between G4 (1.2 ± 0.1 µm) and G5 (1.1 ± 0.2 µm). The axonal diameter was also observed in G2 (0.2 ± 0.1 µm) **(**Table 4**)**.

Concerning the axonal diameters in the N2 nerve segment, no statistically significant difference was observed between G3 (3.1 ± 0.1 µm) and G1 (3.1 ± 0.3 µm). Likewise, no difference was found between G4 (1.0 ± 0.1 µm) and G5, as shown in Table 4.

In G3, the axonal diameter in the N1 nerve segment (2.2 ± 0.2 µm) was smaller than that of the N2 nerve segment (3.1 ± 0.1 µm) **(**Table 4**)**.

## DISCUSSION

Viterbo et al.^6^^,^^8^^,^^9^ introduced a technique for performing end-to-side neurorrhaphy that did not involve any injury to the donor nerve. Many authors subsequently confirmed that both sensory and motor nerves had reinnervated recipient nerves through collateral sprouting, through using this technique.^10^^,^^11^^,^^12^ Furthermore, these results were comparable to those reported using end-to-end neurorrhaphy.^13^^,^^14^^,^^15^ Following this, many techniques were developed in an attempt to reduce the lesions and functional damage to the motor and sensory nerves of the target organs.

In the present study, double muscle innervation through end-to-side neurorrhaphy was created as an alternative to avoid sectioning one of the branches of the deep temporal nerve as well as avoiding the risk of muscle atrophy, which occurred in the technique reported by Labbé et al.^4^

The end-to-side neurorrhaphy was performed 1 cm distally to the sciatic nerve division. Although it was observed that the location of this anatomical site is highly variable in rats, a distance of 2 cm to 3 cm from end-to-side neurorrhaphy to the muscle to be reinnervated was maintained.

The smallest diameter of the axonal fibers was measured. Compared with the larger diameter and area values, this measurement is more reliable, since its magnitude is not affected by the inclination of the razor during transverse sectioning of the fibers.^16^

Despite the sectioning of the peroneal nerve and rotation of both stumps through 180 degrees, with insertion and fixation in the adjacent musculature, a few fibers (1 ± 0.5 axons/mm^2^) were observed in the distal peroneal nerve in G2 after 120 days. This finding was probably from one of the 20 rats in G2, whose distal stump was loose and spontaneous reinnervation occurred.

The fibers in the peroneal nerve segment distal to the end-to-side neurorrhaphy (N1) in G3 had smaller axonal diameters than those in G1. This demonstrates that a lesion occurred in the peroneal nerve, probably while the end-to-side neurorrhaphy was being performed. These findings were confirmed using electrophysiological tests. However, no damage was observed in the cranial tibial muscle in this group, as shown by the weight and diameter results from the muscle fibers.

Nerve lesions during end-to-side neurorrhaphy were also reported by Cederna et al.,^17^ even when meticulous surgical techniques were implemented. These lesions caused acute denervation in the muscle, which was originally innervated by the donor nerve, but without reduction in its contractile power. After six months, no statistically significant difference was observed when body weight, muscle weight and muscle contraction power were compared, which demonstrates that the lesion at the time of coaptation did not lead to functional or structural impairment in the muscle. Other authors have reported a lack of functional deficits in the muscle that was innervated by the donor nerve.^18^^,^^19^

Reinnervation of fibers with lesions was also observed in the axonal diameter in the N1 segment in G3. Higher density and axonal diameter were observed in N1 than in N2 in G3, thus confirming that reinnervation had occurred. However, the origin of the regenerative fibers could not be determined. These findings reaffirm those reported by Isaacs et al.^20^^,^^21^

In a study on reverse end-to-side neurotization, Isaacs et al.^20^ reported findings from groups similar to G3 and G4 of our study. However, they studied female Sprague-Dawley rats for 140 days, while in our study male Wister were studied for 150 days. Those authors created an epineural window in the peroneal nerve at the end-to-side neurorrhaphy site; in our study, end-to-side neurorrhaphy was performed in the intact peroneal nerve, without an epineural window. Another difference between these studies concerned the target muscle. In the reverse end-to-side neurotization, the long digital extensor muscle was analyzed, while for double muscle innervation, we analyzed the cranial tibial muscle. Moreover, we presented the results from G5, in which both the peroneal and the tibial nerve were sectioned proximally to the suture 120 days after end-to-side neurorrhaphy.

Isaacs et al.^20^ reported that no contractions of the long digital extensor muscle were observed when the peroneal nerve was sectioned proximally to the end-to-side neurorrhaphy, although histological analyses revealed immature axons in the epineural spaces of the peroneal nerve. This shows that if the regenerative fibers had originally been from the tibial nerve, they would not have made contact with the motor terminals. However, the authors did not identify the true origin of distally regenerating axons.

In another study on reverse end-to-side neurotization, Isaacs et al.^21^ performed an end-to-end suture proximally to an end-to-side neurorrhaphy. Their findings suggest that regenerative axons from both the recipient and the donor nerve were present. However, once again, there was no quantification of regenerative axons or identification of their origins.

In our study, the results from the electrophysiological evaluation on G4, in which the peroneal nerve was sectioned proximally to the end-to-side neurorrhaphy, revealed that Wallerian degeneration had occurred in the N1 nerve segment, which led to temporary denervation of the cranial tibial muscle and muscle atrophy. Therefore, the tibial nerve was not capable of maintaining muscle innervation after the peroneal nerve had been sectioned.

On the other hand, the electrophysiological test was able to provide confirmation that reinnervation had occurred in only 30 days. The test showed that the amplitude had decreased but latency had been maintained, 30 days after the peroneal nerve had been sectioned proximally to the end-to-side neurorrhaphy. These findings confirmed that electrical stimulation arrived at the cranial tibial muscle through the neurorrhaphy. In contrast, a decrease in amplitude was followed by absence of latency in G5 (denervated) after the peroneal and tibial nerves had been sectioned.

However, regarding the axonal density or axonal diameter results, G4 and G5 were not different. This shows that 30 days was too short a time to see morphometric changes in the axonal fibers. Perhaps a longer time would confirm the findings from the electrophysiological test.

The results from the peroneal nerve analysis in G3 and G4 demonstrated that occurrence of Wallerian degeneration is a limiting factor for axons implanted at the side of a nerve to achieve functional innervation. These findings are in accordance with previous findings from the literature.^20^^,^^21^^,^^22^

Our results did not confirm the findings reported by Furukawa et al.,^23^ in which double muscle innervation was observed after the proximal stump of the hypoglossal nerve had been sutured to the lateral side of the facial nerve with an epineural window, in rats. A suture with six stitches was performed, which may have caused a lesion in the facial nerve. Moreover, those authors reported that synkinesis and facial weight movements occurred in the group with end-to-end suture, while synkinesis was observed in only two out of six animals in which end-to-side neurorrhaphy was performed. Those authors also reported that eight weeks after suture emplacement, the facial nerve was sectioned proximally to the end-to-side neurorrhaphy and the animals presented with a phenotype of facial paralysis. After two to four weeks, they started demonstrating the same synkinesis as in the group that had had an end-to-end suture placed, i.e. innervation of the facial nerve through the hypoglossal nerve started after Wallerian degeneration had occurred.

Furukawa et al.^23^ suggested that the neurons of the intact facial nerve could inhibit sprouting of the hypoglossal nerve, and that nerve terminals would first need appropriate innervation conditions, until denervation of the facial nerve took place. This hypothesis was confirmed by the results obtained in the analysis on G4 of our study, in which muscle reinnervation was confirmed 30 days after the peroneal nerve had been sectioned proximally to the end-to-side neurorrhaphy. Therefore, it can be assumed that, for axonal growth from the donor nerve to the recipient nerve to occur, Wallerian degeneration and muscle denervation are required, so that not double muscle innervation but reinnervation is obtained through end-to-side neurorrhaphy.

Future studies are needed in order to find an alternative that avoids sectioning of nerves, as well as avoiding the risk of muscle atrophy, which occurred with the technique of lengthening temporalis myoplasty.

## CONCLUSION

In this experimental model, double muscle innervation did not occur through end-to-side neurorrhaphy. The tibial nerve was not capable of maintaining muscle innervation after the peroneal nerve had been sectioned, but reinnervation of the cranial tibial muscle was observed after 30 days.
